# The influence of COVID‐19 on modes of governance for climate change—Expert views from the Netherlands and the UK

**DOI:** 10.1002/eet.2042

**Published:** 2022-12-30

**Authors:** Cas Bulder, Iain Todd, Darren McCauley, Mary‐Kate Burns

**Affiliations:** ^1^ Erasmus School of Social and Behavioural Sciences, Department of Public Administration and Sociology Erasmus University Rotterdam Rotterdam the Netherlands

**Keywords:** climate policy, energy transition, governance, green recovery, pandemic

## Abstract

While the world is still in the grasp of COVID‐19, countries are contemplating how to get their economies back on their feet. With a unique opportunity to do so in a sustainable manner, there is an urgent need to revisit the governance of climate change. Opportunities are clearly there: the resurgence in top‐down policies in the pandemic might spill‐over to climate governance; green economic stimuli might cause an increase in market‐based approaches; or an increased focus on solidarity, inclusion and collective buy‐in may drive more inclusive network‐based governance. Using the classic trichotomy of hierarchy, market and network governance, we have analysed the findings of 60 interviews with expert representatives from government, industry and third sector parties in the UK and the Netherlands. Their consideration of the key policies and measures needed to help the transition forward point towards a clear desire for a more hierarchical approach. In addition, mixing the three approaches, especially market and hierarchy, is considered the best way forward.

## 1 INTRODUCTION

The delivery of climate change goals is the most significant dilemma facing the world at his time. It is urgent in time‐scale, global in scope, and comprehensive in nature. It affects all aspects of societal behaviour. A significant body of literature has evolved to assess every aspect of its origins, the barriers which exist to impede its delivery, and the mechanisms available to address those barriers. This includes the study of the modes of governance which are central to the delivery of such a challenging agenda (Bauer et al., 2012; Bednar et al., 2019; Bulkeley et al., 2011; Juhola & Westeroff, 2011; Karin, 2010). However, since its emergence in December 2019, the COVID‐19 pandemic has dominated the world's attention and resources. While the pandemic threatens people's health, the indirect effects of lockdowns and social distancing are no less devastating. The disruptions of social and working life have caused economies to plunge into the worst recession since the Second World War (The World Bank, 2020). International bodies, national governments, and society in general are all struggling to reconcile the demands of the two competing challenges of climate change and COVID‐19.

The response to date has been understandable—to seek opportunities to develop an integrated approach which can address both challenges. For example, international bodies are calling for a long‐term economic stimulus (Commission, 2020; OECD, 2020). Individual governments are rebooting their economies to stimulate employment opportunities and green growth (IEA, 2020). But there are risks that the immediate demands of COVID‐19 could eclipse the less apparent needs of climate change. So, it is paramount to discuss how the handling of these twin challenges—and their interaction—should take form. Opinions are divided. There are those who argue that the disruptions caused by the global pandemic may not harm the governance of climate policy (Dupont et al., 2020). Others note how countries are adopting tailored approaches to the subject (ClimateAnalytics, 2020). Commentators postulate that nationalist approaches are increasing and international cooperation is diminishing (Elias et al., 2021; Williams et al., 2020; Woods et al., 2020). These emerging developments inevitably influence the governance of the climate change agenda (Chapman & Tsuji, 2020).

Governance is an ever‐contested subject. Contemporary climate governance is made up of diverse variations of policy mechanisms reflecting hierarchical, market or network approaches (or a mix of these). Our approach is founded on such a tripartite structure of modes of governance, while recognising that hybrid combinations of these three modes are certainly possible and often provide the most appropriate description of the situation on the ground. We test the classic mode of a top‐down hierarchical mode of governance, which has been deployed by governments towards the pandemic by imposing lockdowns and related measures. This has shown that top‐down policy can change behaviours (Hale et al., 2021), raising the question of whether a more top‐down approach would also benefit climate policy. On the other hand, these developments may cause an increasing reliance on government intervention and regulatory governance, and this could distract other elements of society from their responsibility to deal with the climate and energy transition.

With the opportunity provided by COVID‐19 to speed up climate policies, we need to determine how governments, industry, and the third sector frame the transition in the light of the pandemic and how they believe the governance of climate change should be conducted in the future. To do so, we focus our research questions on a comparative study between the Netherlands and the UK in this regard. Our approach is therefore clearly at the national level, while recognising that such an approach could equally find application at the international level or at the level of the community. Both countries have presented similar responses to the pandemic in terms of lockdown and associated measures (Hale et al., 2021). Both are committed to the Paris Agreement and have taken up action to meet this climate commitment prior to the pandemic.

Taking a qualitative approach, we conducted 60 elite interviews with key stakeholders from government, industry and the third sector involved in shaping and delivering the energy transition in both countries. We analyse their views on how they perceive the governance of the energy transition and what it should be ideally. In doing so, our conceptual model is framed around four questions. First, has the pandemic made the hierarchical mode of governance more important to the delivery of the energy transition? Second, if so, is there a consequential weakening in the role of the market mode of governance? Third, how has the pandemic affected calls for widespread societal involvement in a networking mode of governance to the same end? And fourth, do our interviews reveal an emergence of hybrid governance as a favoured mode? These four questions provide the structure of the subsequent sections of this article, which aims to contribute to literature on climate change governance and provide valuable insights in the handling of the energy transition in a post‐COVID world.

## 2 LITERATURE REVIEW

The definition of the term governance has received significant academic attention, with a myriad of interpretations available (Bernauer & Schaffer, 2010; Breakey et al., 2016; Karin, 2010). But no single universal definition of governance has been formulated. Evans (2012) recognises governance as a process of steering society, with a critical role to play in co‐ordinating disparate voices and securing collective action to advance to a more sustainable future. However, according to Hillman et al. (2011), past attempts to conceptualise modes of governance have sometimes led to more confusion than clarity.

Furthermore, it is possible to differentiate between governance at various levels of the public sector—international bodies, nations, and communities. Gibson et al. (2000) notes that sustainability governance can be defined as a multi‐level and multi‐scale challenge. Many references relate to the global scale, choosing to examine sustainability governance through the lens of international bodies. For example, Bernstein and van der Ven (2017) concentrates on the role of the United Nations, describing an approach to governance which is termed an act of orchestration, with significant use of soft modes of influence. This links to the concept of polycentric governance—advocated by Ostrom (2010) and Cole (2011)—in which a sub‐global approach is encouraged, involving multiple centres of semi‐autonomous decision‐making. Conversely, some authors have examined the application of modes of governance at the city scale (Palm et al., 2019). On the issue of level of governance, we clarify that our study concentrates on modes of governance operating at the national level.

In considering the variety of modes of governance, there is widespread acceptance of a tripartite classification of the subject involving hierarchical, market, and networking modes of governance. Such models discussed in the literature range from state‐centred (Bauer et al., 2012; Bell & Hindmoor, 2009) to corporate‐centred (Jordan, 2008; Mallin et al., 2013) and society‐centred approaches (Bell & Hindmoor, 2009; Rhodes, 1996). These three modes are discussed individually in the following subsections 2.1, 2.3.

In doing so, it is important to be aware of two trends in the literature. The first is that there has been over time a move from top‐down governmental action to an increasing reliance on the interaction with non‐state actors to produce public value (Bell & Hindmoor, 2009; Klijn & Koppenjan, 2016; Kooiman, 1993; Osborne, 2006; Rhodes, 1996). They note that the increasing significance of complex societal issues renders top‐down government unable to provide solutions on its own, requiring support from the market and from networks. The second trend is that rather than consider the operation of a mode of governance in isolation, there is increasing study of modes acting in combination—hybrid modes (Lowndes & Skelcher, 2002; Osborne, 2006; Rhodes, 2012; Torfing, 2012). This aspect is considered further in subsection 2.4 below.

### 2.1 Has the pandemic made the hierarchical mode of governance more important to the delivery of the energy transition?

The classic form of governance comes from a top‐down hierarchy, under which compliance is achieved through a mixture of incentives and penalties determined and implemented by government (Kooiman, 1993; Rhodes, 1996). It reflects the authoritative actions of state‐actors. They are characterised using policy instruments—laws and regulations—which are secured by enforcement and which directly control behaviours (Bell & Hindmoor, 2009; Molenveld et al., 2020; Torfing, 2012). Other governmental powers include the use of the fiscal system, the deployment of financial research support, and the use of government communication systems to promote behavioural change.

A hierarchical approach can be justified on two grounds, as put forward by Jänicke (2008). The first is the traditional market‐failure argument, under which it is postulated that policies cannot be delivered on the basis of the market alone. The second is the perception of the environment as a public good, which should therefore be protected and enhanced by the public sector. But there are also critics who advocate that hierarchy cannot operate alone. Blanc (2009) advances that the concept of a government‐only model is a fiction. Giddens (1990) describes the relationship between the public and private sectors as characterised by increasingly complex webs of interdependence, requiring an altogether more sophisticated and inter‐related model. In responding to such a trend, commentators are divided on whether a changing role for government represents a loss of power, or is simply a means of achieving results through other means. In other words, such changes may represent either a threat or an opportunity, or both.

### 2.2 Is there a consequential weakening in the role of the market mode of governance?

Hierarchy is often combined with markets. Not everything can be governed through regulation and control. Hierarchical government is complemented by market governance, which uses economic principles to influence behaviour. Government may not only interfere in the ‘free’ market to correct its imperfections but can also use the market to steer towards preferred behaviour. With positive and negative incentives that affect the consequences of actions, behaviours are more indirectly influenced (Howlett, 2009; Molenveld et al., 2020).

Some scholars even argue that the distinction between market and hierarchical mechanisms is blurred (Wang et al., 2018). As governments excessively intervene in markets with cap‐and‐trade mechanisms such as the emissions trading system, carbon taxing and subsidies, market mechanisms can be viewed as being applied hierarchically. This combination of market and hierarchical intervention is commonly used in national and supra‐national climate governance (Markantoni, 2016; Miao & Li, 2017). The market‐based approach stems from the definition of the climate crisis as market failure in which the government has a critical role to play (Dryzek, 2005; Lo & Francesch‐Huidobro, 2017). But despite its popularity, the combined hierarchy/market‐based approach is considered to be inadequate as the sole governance mode to address the climate crisis, as it cannot invoke the full collaboration and commitment required for addressing this problem (Lo & Francesch‐Huidobro, 2017; Lowndes & Skelcher, 2002).

### 2.3 How has the pandemic affected calls for widespread societal involvement in a networking mode of governance?

Steering using market principles may not always provide the intended or required behavioural changes (Torfing, 2012). Following the imperfections of hierarchical and market governance, networks have provided a third mode of influencing societal behaviours. In networks a multitude of interdependent actors collaborate voluntarily towards a shared goal, helping to produce or deliver public goods and services (Jessop, 2002; Kickert et al., 1997; Klijn & Koppenjan, 2016). Within these networks, we can make a distinction between those which are state‐centred and those which are society‐centred. In state‐centred networks, government is a pivotal actor, sometimes even mandating the network (Bell & Hindmoor, 2009; Popp et al., 2013). In society‐centred networks, government is just one actor, if an actor at all. We usually associate these networks with self‐organisation (Rhodes, 2012). However, while government might not always act from a hierarchical position, these networks still operate in the shadow of hierarchy (Bell & Hindmoor, 2009; Provan & Kenis, 2008).

In climate policy, we often find networks in international agreements, which may be national (Ottens & Edelenbos, 2018) or supra‐national (Hoch et al., 2019). Most recently, a surge of network governance has been witnessed, particularly in urban adaptation strategies (Covarrubias et al., 2019). While a lack of coordination between regional and national hierarchical mechanisms can cause a hindrance, networks can coordinate stakeholders across sectors and dip into local knowledge required by robust adaptation policies (Hanssen et al., 2013). But it is necessary to consider the context in which these networks operate as demonstrated in recent work in Moldova (Ciobanu & Saysel, 2020). Networks are prone to stagnation, corruption, and elite capture (Edelenbos & van Meerkerk, 2016; Nochta & Skelcher, 2020). Sometimes, an absence of hierarchical steering results in performance or legitimacy problems (Bauknecht et al., 2020; Juhola & Westeroff, 2011; Nochta & Skelcher, 2020). Scholars often point towards anchoring networks in democratic institutions which—alongside regulation—can help to solve these issues (Klijn & Koppenjan, 2016; Sorensen & Torfing, 2009).

Thus, while networks may have suitable properties for solving complex societal issues, they are not without problems. Some studies even find hierarchical arrangements to be more effective (Cadman et al., 2017; Goldthau & Sitter, 2019). Though acknowledging that private sector involvement and stakeholder consultation are crucial for raising efficiencies and legitimacy, the public responsibility tied to hierarchical steering may cause higher implementation rates (Mees et al., 2013). When calling for a stronger regulatory government, however, it is generally in combination with markets, networks, or both (Dirix et al., 2013; Gaspari & Lorenzoni, 2018). The sole use of regulatory mechanisms lacks the flexibility and cross‐cutting properties needed for the adequate mitigation of complex societal issues.

The differences between the governance modes are grounded in the advocated level of government intervention. While some recognise that government has the necessary capacity and legitimacy to drive climate action (Kaswan, 2018), others would like to see a less prominent role for government while pointing towards the more suitable characteristics of network governance (Hanssen et al., 2013; Luthe et al., 2012). The multi‐actor setting of networks allows for broad co‐operation, joint knowledge building, and the use of collective resources. This makes network governance particularly suited for solving complex societal issues (Klijn & Koppenjan, 2016; Ostrom, 2010).

### 2.4 Do hybrid approaches emerge as a favoured mode?

This literature review has provided an account of three different governance modes in contemporary climate and energy policy, namely hierarchy, market, and networks. Considering the pros and cons to each of the modes, consensus in the literature points to no single mode that can be the exclusive generator of adequate climate policy. It is thus in hybrid governance, where one or more modes complements the others, where the most effective policies are found. Pahl‐Wostl (2019) argues that hybrid governance systems with synergistic interplay between the three classic governance modes are essential for dealing with complex challenges (in their case, water management).

The concept of ‘modern governance’ is championed by Kooiman (1993), advising that contemporary forms of governance have sprung from increasing interactions between public and private actors, setting out three essential characteristics of co‐operation, continuous interaction, and joint agreement on rules and norms. While Lange et al. (2013) prefer ‘real world governance modes’, described as practical combinations of hierarchical and non‐hierarchical approaches to sustainability governance.

But which mode of governance should have the primacy in this hybrid mode remains unclear? While a recent study shows a preference of non‐governmental actors for a strong, regulatory government (Molenveld et al., 2020), the desirability of such a resurgence is questionable as this may distract industry and societal actors from their responsibility to play their part in the climate and energy crisis. Considering the developments around climate and energy policy following the global pandemic described above, we argue it is crucial to explore further how the three governance modes should be employed.

## 3 METHODS

The first methodological decision in framing this research was the selection of the two case study countries. The selection of the Netherlands was determined by the funding call of the Dutch Research Council (NWO), but it did provide a valuable example of a typical western European nation tackling the combined pressures of the energy transition and the COVID crisis. Its approach to the former can be described as in the leading cadre, with a high degree of awareness and commitment to dealing with the issues involved. Its approach to COVID—at that early time—was representative of many European nations, within the decision‐making framework of the EU. The addition of the UK as the comparator case study was based on both the similarities and differences between these two nations, which would lead to valuable observations. Both share a broadly similar economic, social, and political history. Both are party to the Paris Agreement (United Nations, 2015), have active climate change programmes prior to the COVID crisis, and are/were net contributors to the European Community budget. Both have very significant fossil fuel industries. But the way forward for the UK lies outside the EU, and this could lead to points of policy difference. This project therefore provided an excellent opportunity to examine the similarities and differences in how their responses to the COVID crisis impact their energy transitions.

To reach an in‐depth understanding of these views we have taken a qualitative approach focussed on 60 elite semi‐structured interviews. After identifying 15 key stakeholders per country based on their experience and relevance in the climate and energy sector, each organisation was interviewed twice—in July and October 2020—to track potential changes in perceptions. Interviewees were selected on the key parameter of experiential relevance, in the categories of government, industry and third sector to provide a varied range of state‐ and non‐state actors. As a result, organisations ranged from government officials (national and municipal) and multi‐nationals to NGO's and trade unions; these are shown in Table 1 below. We note that while the numbers are matched between the two countries, there are caveats that the Dutch responses were more dominated by government and third sector, while the UK responses were dominated by industry representatives. Most interviewees were in the age range of 45–60, having reached relatively senior positions in their organisations; but there were exceptions such as the senior school representative of the Dutch students' union. The gender mix was approximately 25% female; this mirrors the gender mix in senior positions in the energy industry in the Netherlands and the UK, and so was judged acceptable.

**TABLE 1:** Make‐up of stakeholder interviews

Stakeholder	All	NL	UK	Govt	Industry	Third sector
Number of interviews	60	31	29	23 (NL 15, UK 8)	19 (NL 3, UK 16)	18 (NL 13, 5)

In conducting the interviews, we completed a full ethical and risk assessment. Participants were informed of the voluntary and confidential nature of the interviews, allowed to ask questions, withdraw at any moment, and asked for consent prior to their participation. Consent was also asked for the recording of audio and visual material used to transcribe the interviews and forthcoming quotations. Due to COVID lockdown requirements, all interviews were conducted virtually.

The research questions forming the basis for the interviews were structured around two themes. The first theme relating to the effects of the pandemic in both countries. Questions concerned the identification of vulnerable social groups and the impacts of COVID on the energy transition. The second theme aimed to identify which policy mechanisms were desired to reduce the impact of COVID‐19 on the energy transition in these countries. Participants were invited to consider the full range of regulatory, non‐regulatory, and more network‐based policies.

For analysis, we coded all 60 transcribed interviews using the software program Atlas.ti. The basis for this analysis was a detailed coding tree, which was verified by three different researchers for triangulation. The coding for delivery mechanisms is shown in Table 2 below, which is structured according to the three modes of governance which have been reviewed in section 2. Table 2 also records the distribution of the 113 key observations within the coding. The key interview findings in each modal category are described in more detail in the following section 4, which includes further information on which stakeholder groups contributed these observations. The implications of section 4 findings are then discussed in section 5.

**TABLE 2:** Coding of interview transcriptions on mechanisms needed to address climate change

Mode of governance	Coding tree	Number of key observations
Hierarchical	Fiscal policy	11
	Investment policy	28
	Business policy	9
	Communications	9
	Institutions	8
Market	Industry	10
	Technology	0
	Finance	19
Network	Societal	9
	Municipalities	4
	Labour	6
Total		113

**FIGURE 1:**
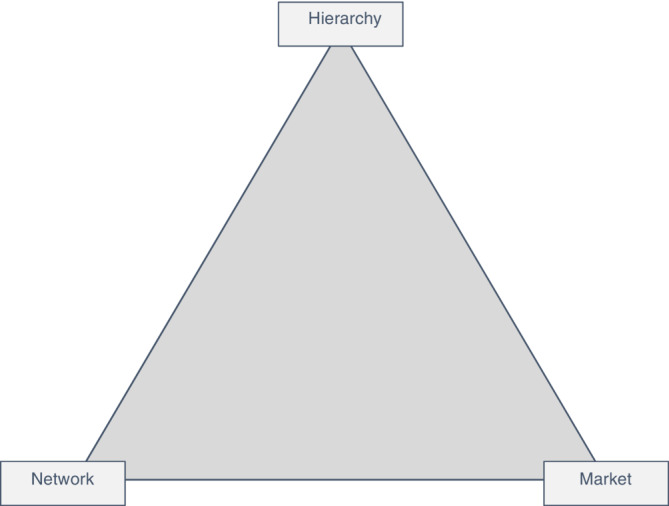
Hybrid governance models for pandemic responses. *Source*: Authors

**FIGURE 2:**
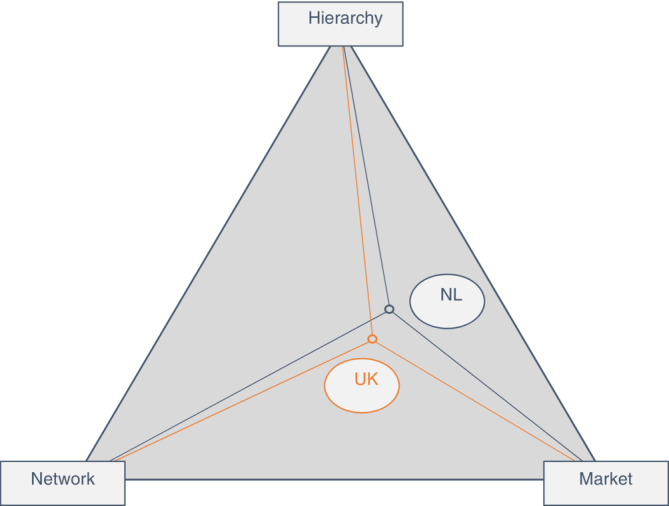
Hybrid governance models for pandemic responses—The UK and Netherlands. *Source*: Authors

One final element of our method relates to the post‐analysis presentation of results. We have prepared a graphical representation of the three main modes of governance as the apexes of a triangle, as shown in Figure 1 below. Such a representation introduces the possibility of locating hybrid combinations of the three modes within the triangle. This was indeed carried out by means of a simple numerical summing of observations relating to each mode, thereby allowing—for each country—a plotting of that country's position within the triangle, reflecting the relative importance of the three modes of governance. This plot is included as Figure 2 in section 5.2 of the Discussion.

## 4 FINDINGS

We now report our interview findings on the different approaches to governing an energy transition. They are structured here according to the three principal forms of governance assessed in the literature review, namely hierarchical governance, market governance, and network governance. Comments were balanced between these three forms, with most expressed in terms of market governance. In a fourth subsection 4.4, observations are made on a hybrid approach to governance, which involves combinations of the three basic types. This reflects the fact that the classifications of governance do not truly act in isolation—there are grey areas between them. Nevertheless, the concepts involved in individual forms of governance provide a useful framework against which to assess the observations made by our interviewees.

### 4.1 Has the pandemic made the hierarchical mode of governance more important to the delivery of the energy transition?

The classic form of governance comes from a top‐down hierarchy, under which compliance is achieved through a mixture of incentives and penalties determined and implemented by government. This is often achieved through regulation. While this approach suffers from the disadvantage of the length of time needed to introduce legislation—partly due to the need for consultation—it does enjoy the benefits of universal application and ease of enforcement. The need for a leading role from government was recognised by one UK industry interviewee:

‘I get the reason for government's desire for a free market. But there is an awful lot of understanding – particularly from the financial crash of 2008 – that you must have a reasonably regulated free market. Otherwise, you end up with perverse outcomes. The world needs to have enough governance behind it to achieve net zero’.

This was supported by several interviewees who emphasised that policy commitments by government must be long‐term, and this was an essential component of effective governance. They criticised their experience of ‘stop‐go policies’, ‘a blame game’, and short‐termism. In the words of one UK third sector interviewee:

‘There's lots of well‐intentioned individuals and initiatives but where is the consistent long‐term policy for delivering it?’

Interviewees from both UK industry and Dutch industry also addressed the need for policy stability:

‘What will support investors is the stability around policies, that make them comfortable that the investments they put into various locations will be secure over the lifetime of the project, the lifetime of that asset, and the return on investment’.

‘If you make a stable investment path, no matter what happens – in economic downturns or pandemics – we are going to stay on that path’.

A seemingly tailored response to these observations was provided in an interview with a representative of the Dutch Government, who also referred to policy stability:

‘So, in the end you want the climate policy to be stable in such a way that the rate of change in the transition towards the goals is not dependent on – for example – sudden price drops in the energy markets. You want to design a policy in such a way that it is resilient to these developments. If you want to summarise it, I think the solution is more strict regulation for implementation of the targets and underlying measures’.

And on a specific example—of increasing the number of electric vehicles—another representative of the Dutch government considered that:

‘This would be easier if there is strict regulation in line with the new EU targets. If you do not have these targets at the EU level, you have to come up with fiscal subsidies, which can have all kinds of side‐effects’.

This last observation shows first that regulations can be set at diverse levels of public administration. This argues for consistency between those levels, and the need for effective consultation and co‐operation between them. It second sets out the possible use of fiscal incentives as an alternative to regulation but noting that this can lead to adverse side effects. But, in contrast, most of our interviewees favoured the use of market signals of that type, as will be described in the following sub section. Another interviewee linked the hierarchical approach to the need for effective implementation, monitoring, and enforcement, considering it important to:

‘.. include a monitoring role which would research the progress towards a just transition, and how well all groups in society are getting along’.

We find a significant body of comment in favour of strong and effective governance by the two governments, acting in hierarchical mode. But, in contrast, most interviewees went further, recognising that the force of the market was an essential component to governing the energy transition, so involving the expertise and resources of the commercial sector.

### 4.2 Is there a consequential weakening in the role of the market mode of governance?

While some interviewees foresaw the need for a top‐down regulatory control of policy development, the majority commented favourably on achieving objectives through market governance. This encompassed several mechanisms: the use of investment on green infrastructure (which might be public or combined public‐private); the deployment of government subsidies; offering incentives or disbenefits through the taxation system; and maintaining research investment. The nature of such mechanisms does indeed blur the distinction of market and hierarchy.

The issue of investing in green infrastructure dominated the responses received in interview. This was a vital feature before the COVID crisis, and it was considered even more so as part of the economic recovery post‐pandemic. All saw a considerable synergy between the two tasks. Important questions were raised on—for example—the appropriate division of responsibility between public and private investment. One interviewee in the UK government summarised the issue as follows:

‘There is an opportunity to think. We can expedite some of this transition now, use the opportunity of the need and the demand to provide an economic stimulus, and to frame that stimulus in a way which brings forward and locks in much more quickly some of the transition in the energy sector’.

This demonstrates the intimate connection between hierarchy and market. The interviewee considers this is the way to deliver long term change, but with a market first approach. This was most pronounced by our UK interviewees. We reflect on this further when considering the hybrid governance model below. This view was supplemented by two UK industry interviewees, who argued first that:

‘If you think about the big energy companies, they really have a lot of money behind them. They have the power to take some of these ideas to the next stage and maybe try to commercialise them. That doesn't necessarily need government funding’.

But on the other hand:

‘The public sector should invest in some projects that the private sector would not invest in. But they should be very careful which they are. If a project is not viable in the private sector, the public sector will not make it viable – that is not where their skillset lies. And investment in such projects will draw resources away from other viable improvements in society’.

The connection between market and hierarchy is observable most in reflections on fiscal incentives. The role of taxation was frequently referred to as a possible mechanism to assist effective market governance. In the words of one UK industry interviewee:

‘Taxation will inevitably have a part to play to discourage people or to make it very expensive for certain types of journeys and certain types of travel’.

Whilst more pronounced in our UK interviews, the need for specific taxes was addressed by several of the Dutch third sector groups:

‘I think it's very important to move forward on carbon taxation, including carbon pricing and carbon compensation schemes. This is absolutely necessary for the energy transition’.

‘Make it more expensive to drive a car, especially in heavy traffic, paying according to both distance and degree of congestion. The additional taxation raised should be used to lower the taxation of incomes. This would be a progressive development’.

Taxation is at the centre of climate governance debates, but the provision of other methods of market support—such as subsidies and financial grants—also featured. According to an interviewee from the UK government:

‘New technologies are expensive, and they are not going to happen without some form of government support, in view of the risks and the costs involved. Our job is to understand what would happen in the absence of government support, where we can best intervene to bring that forward. There definitely is a role for this’.

The need for a careful and targeted analysis was supported by UK industry:

‘Investment isn't needed in certain technologies – such as heat pumps – but rather in the associated business models. These should allow people to take up new technologies at little or no upfront cost’.

One Dutch third sector interviewee linked the role of financial incentives to the need for behavioural change:

‘I think that financial incentives will be the most successful instrument to produce behavioural change, because I fear that based on good intentions alone, the green and social transitions will not take place or develop that rapidly’.

The importance of investing specifically in research was mentioned by several interviewees, including the following from the Dutch government:

‘I would focus on green investments, and – as shown in a previous crisis – if you invest less in research, it will not be beneficial. So, one of the things we plead for …. is to keep investing in innovation because that is something you particularly need in a crisis’.

### 4.3 How has the pandemic affected calls for widespread societal involvement in a networking mode of governance?

There was an appetite among all stakeholders that the responsibility for change should be more widely spread than just government and industry, and this was particularly true when the transition under consideration involves action at the community level and at the individual level. This was recognised among many interviewees. In the words of one UK industry interviewee:

‘Although the COVID crisis has made people physically further apart, it has brought a lot of communities closer together, in a way they had not been before. Community cohesion is something that you could use positively in the energy transition. So, one trend we are looking at ‐ emerging even before the crisis but which we think could be accelerated ‐ is the concept of energy communities’.

This was echoed by a Dutch third sector interviewee, who believed strongly in participatory power:

‘The government should not just act top‐down, such as issuing regulations. Society is such a diverse mix of people and situations, there is no one size which fits all. It is therefore a very careful process to arrive at the best solution for all parties’.

Beyond this, network governance modes appear below national level and outside those making policies. In these remarks, ‘community’ and ‘all parties’ indicate a dual frustration that top‐down iterations of climate governance are letting down local people, and groups outside formal decision‐making processes. Network governance is therefore a response to formality.

A further UK industry representative considered that:

‘I personally believe that behavioural changes are required as a priority, above and beyond some of the investment piece’.

There was however recognition that such an approach involves important challenges. A UK industry interviewee considered that communities might lack the expertise to take on a more substantive role:

‘One of the key barriers we have is community capability and community infrastructure. … Local energy systems are going to need not just bodies, but also expertise’.

Moving beyond formal government or market mechanism was not the only preoccupation of interviewees. Information, communication, and transparency are key concerns that network governance can tackle. This points to such issues, alongside rejecting formal decision‐making, as drivers of network governance. A lack of information was especially identified by two UK government officials as related to the effectiveness of communications with communities.

The first addressed the need for better collaboration between industry and communities:

‘I just don't think the energy industry does a very good job of communicating with citizens. And that is going to be a problem if we are trying to bring about an energy transition where consumers are much more at the centre of it. If the way we communicate does not change, that's going to be a real problem’.

And the second was more focussed on home ground:

‘There is very limited communication from government with consumers themselves about their use of energy and about the options open to them’.

One further UK industry interviewee focussed on the important role that could be played by cities in a scenario based on networked governance:

‘Cities can have different politics from the whole country, and so a lot of the drive to decarbonisation is provided by cities taking the leading role. Regardless of their national plan, they are making their own ambitious goals. And such cities are joining together with other cities in Europe to share their ideas’.

We found an overall consensus for an increased prominence on societal behaviours, the need for motivation and involvement at the grass roots level, and communication campaigns to promote such involvement. These are in response to the gaps found in formal hierarchical and market governance modes, identified above as information, communication, and transparency. There was recognition of the key potential role for cities and communities. Existing research on network governance is too focused on investigating cities only. Our findings also point to non‐urban communities as evolving players in network governance. We see accountability, data‐driven, climate networked governance to be both urban and rural. We call for further research on this issue.

### 4.4 Hybrid approaches to governance

In the preceding subsections, we assessed the governance of the energy transition in the context of three perspectives—hierarchical governance, market governance, and network governance. These are useful models from which to analyse complex societal processes. But reality is more nuanced, and all three were interwoven in the interview discussions. These interviews pointed consistently to an energy transition that demands a balanced response from multiple perspectives of ideal governance arrangements. There was a recognition not only of inter‐connection, but also of synergy—that with appropriate management, the combination of the three approaches could add up to more than the sum of the three constituent parts.

Many interviewees commented on the desirability of mobilising the combined resources of government and industry. Hybridity in governance is built on the hierarchy and market approach; our research confirms this in both the Dutch and UK context. Interviewees expressed a frequent desire to see the full range of hierarchical and market‐based mechanisms used in the pandemic recovery. Responses emphasised that both sets of national policymakers adopt new combination of such mechanisms to help speed up the transition. In the words of a representative of a Dutch multi‐national:

‘Governments will become more important in the years to come. The whole “leave it up to the market and it will be alright” is something that people doubt at this moment in time. There is a big outcry for more government intervention. And it is not only from citizens, but from the companies themselves. So, I think there will be a lot of government money flowing into the system, but also of course the government will not give it for nothing. They will have their demands as well, in terms of sustainability, in terms of future‐proofing, all that kind of thing’.

These thoughts were echoed by a Dutch interviewee from the third sector:

‘If you have all these state investments, they can come with requirements of sustainability targets. So, if the government pays a lot of money to these companies, they can also have their demands of greener production’.

However, we also find evidence throughout our interviews of a need to integrate network governance, in discussions on the hierarchy‐market relationship. This was most often the case for third sector participants, but not exclusively. We raise an example from one UK industry interviewee who considered, for example, that there were risks in involving big industry in community projects:

‘As soon as you get big energy companies involved – or lots of different players requiring a slice of the profits – the end users generally are not the ones that get the benefits. But energy communities bring the decision‐making more into the community's hands and the profits back to the consumers’.

This all argues for a composite approach to modes of governance.

## 5 DISCUSSION

We now discuss the implications of the findings reported in the preceding section, against the background of the literature review reported in section 2. In doing so, we adopt a broader perspective, seeking to examine any differences in the views of the two countries and the views of sub‐sectors, in relation to how the pandemic might be affecting the preferred mode of governance of the energy transition. In the following four paragraphs (subsection 5.1) we consider how our findings on the four questions extend or contradict the findings of the literature on this subject. We then consider in subsection 5.2 a comparison between the findings of the two countries. Here we introduce at Figure 2 a graphic representation of the three classic modes which also serves to show how hybrid modes and differences between the two countries can be demonstrated.

Before doing so, we repeat an earlier point that our approach to modes of governance is considering the national level. Our interviews did include two representatives of communities (Rotterdam in the Netherlands and Aberdeen in the UK) and these emphasised the need for co‐ordination, consistency, and effective communication between different levels of public administration. This supports observations made by Ottens and Edelenbos (2018). We repeat also that our approach does not address the urban ‐rural interface, as addressed by Hughes et al. (2020).

### 5.1 Discussion of four key questions

Beginning with consideration of how the pandemic is impacting on hierarchical modes of governance, we found first a strong demand from all quarters for long‐term government policy set by the centre. This supports the advantages of a hierarchical approach in delivering sustained change, as set out by Cadman et al. (2017), and also a preference among non‐governmental actors for a strong regulatory government, as found by Molenveld et al. (2020). This desire extended into the fiscal role of government, as evidenced by Markantoni (2016), but noting also that fiscal policy can have adverse impacts as noted by work on governance in China undertaken by Miao and Li (2017). Finally, we found a strong support for government to communicate clearly and authoritatively with all sectors, which coincides with findings in Gupta and Mason (2016). We therefore report multiple reasons for the reinforcement of hierarchical governance, with findings that this is being delivered to a greater or lesser extent.

The diverse market support mechanisms identified—investment, subsidies, taxation, and research—were considered vital to motivating the commercial sector to assist with the delivery of the energy transition. The significance of taxation supports the approach of Gaspar and Amaglobeli (2021), who argue that while taxation in climate change literature is too often placed as a government top‐down mechanism, it is more appropriately a way to view market governance in action, underpinned by hierarchy. Government may be the immediate benefactor, but the way the market is constructed, developed and implemented will determine the success of carbon‐based taxation (Geroe, 2019; Goulder, 2013). There was also significant counsel that such support had to be well‐targeted, proportionate, and delivered in a timely manner; the importance of effective implementation coincides with the views of Mees et al. (2013). The role of government may be irrefutable in climate market‐based governance. But market governance responses prioritise economics, finance and infrastructure above the power of top‐down government regulation.

Turning to network governance, there was strong recognition of the need for bottom‐up action involving participatory power, while accepting in parallel the importance of hierarchy. This supports the views of Covarrubias et al. (2019), Ottens and Edelenbos (2018) and Hanssen et al. (2013) on this point, and indeed the findings of Rhodes (2012) that policy‐makers are disillusioned with hierarchical and market approaches in isolation. We report emphatic support for a supporting role for network governance, in support of Lo and Francesch‐Huidobro (2017) and Molenveld et al. (2020). We find also a demand for improved data‐management on societal issues between governmental and community actors, as noted by Hughes et al. (2020) in calling for a new era of accountability through data‐driven urban networked governance.

Finally, the interactions between the views expressed on the three modes of governance inevitably leads to the conclusion that in the real world it is not possible to view the individual modes in isolation. This reflects the views of Cadman et al. (2017), Goldthau and Sitter (2019), and Mees et al. (2013) who advocate that a composite hybrid approach is most representative of society in action. This leads to our graphical depiction of such composite modes, as described in the following section.

### 5.2 Comparison of modal priorities between the two countries

As described previously in section 3, we have prepared Figure 2 below using a simple numerical summation of observations on each mode, based on our coding of the entire interview set. It shows a differing level of commitment to network governance among our Dutch and UK participants. The Dutch interviewees in industry, government and the third sector focused more on the need for greater integration of hierarchical and market solutions. They considered the ideal governance solution for managing the transition to be less dependent upon network governance mechanisms. Both national contexts showed a lack of appreciation for network governance as the most important constellation for achieving an effective transition out of the pandemic. This is a surprising conclusion as it is not in line with existing research (Bauknecht et al., 2020; Ciobanu & Saysel, 2020; Covarrubias et al., 2019; Nochta & Skelcher, 2020) which places more emphasis on network governance. In short, hybridity between the three governance mechanisms is clear, but to varying extents depending on national context. We encourage future research to investigate this in more detail, especially as the transition out of lockdown accelerates.

## 6 CONCLUSIONS

So, the consensus of interviewees would seem to support a hierarchical and market combined governance model with more inclusive, networked form of governance to deliver the energy transition in these countries. A hybrid combination involving of all three forms of governance is evidently the dominant view of our interviewees. The question remains what the ideal balance is between each. This is where we find a nuanced difference between the countries in focus. We present below the ideal types from our interviewees based on national context and reflect briefly on their implications.

The Dutch and UK experiences amid the pandemic revealed a strong preference among the public, private and third sectors for hierarchical and market governance modes. This confirmed our first expectation as set out in the introduction. The interventionist approaches taken by both governments is at least maintaining, if not encouraging, further belief that regulatory approaches are critical for a sustainable recovery. Far from leading to a pessimistic view, high levels of investment into national economies are spreading further demands for wholesale green public financing. A consensus emerged that the pandemic offers a unique opportunity to ride the wave of intervention. Even industry leaders in both nations called for more government support. The third sector in both countries recommended a wider array of regulatory market‐based mechanisms such as taxation and subsidies. In contrast to existing literature (Bauknecht et al., 2020; Nochta & Skelcher, 2020), our findings suggest that the hierarchical mode might thus become more important for governance research.

By contrast, network governance was less pronounced than expected. There was widespread consensus to support empowering community energy projects and sub‐national networks. From industry participants especially, their ideal vision of governance modes includes more integrated connections between key actors at the national level. Network governance was therefore present in our participants' depiction of governance‐based responses, but it was less prominent than the other two modes. It is possible to conclude from this that COVID‐19 has suppressed the networking element of governance, highlighting that this has always been a less well‐established component of a composite approach to governance. It was also here that we find the most significant variation in terms of nationality. There is a recognition of the success played by sub‐national and community‐based projects in UK, but this was less clear in the Dutch case. A second trend in network governance differences is the perception of local government, with Dutch interviewees articulating a local hierarchical approach, whereas UK interviewees expressed the significance of locally networked third sector activities. Nonetheless, we conclude that a move away from network governance thinking is clear, at least in the short term during the pandemic.

COVID‐19 will have long‐term effects on how policymakers view ideal governance modes. From large scale public investment to taxes, subsidies and regulation, the state has rarely been so prominent in our lives. This will have implications for what policymakers, and society at large, consider are the best ways to self‐form in the face of a crisis, whether it be health or climate. Network governance will continue to be relevant in climate debates. Bottom‐up community action, whether it be rural or urban, place‐specific or trans‐national, is a key mobiliser for the transition. But the state, emboldened by its new‐found market ambitions for now at least, may experience a renaissance in climate governance debates. We need future research to expose the hybridity balance of climate governance systems, and where lie the priorities for key stakeholders.

Our study is not of course without its limitations. We have studied only two countries in western Europe, and different conclusions would result from say studying nations in the developing world. We have concentrated on the national level of governance rather than that of international bodies. We have relied on expert interviews, rather than adopting a large‐scale survey of citizens, which again could have resulted in different perspectives. We have not addressed the rural–urban divide. All that said, our findings provide a valid and valuable body of evidence relating to the impact of COVID‐19 on the energy transition in these two countries.
